# UHPLC-QTOFMS-Based Metabolomic Analysis of the Hippocampus in Hypoxia Preconditioned Mouse

**DOI:** 10.3389/fphys.2018.01950

**Published:** 2019-01-10

**Authors:** Wen-Ting Liao, Jie Liu, Si-Min Zhou, Gang Xu, Yu-Qi Gao, Wen-Yuan Liu

**Affiliations:** ^1^Key Laboratory of Drug Quality Control and Pharmacovigilance, Ministry of Education, China Pharmaceutical University, Nanjing, China; ^2^Institute of Medicine and Hygienic Equipment for High Altitude Region, College of High Altitude Military Medicine, Army Military Medical University, Chongqing, China; ^3^The Key Laboratory of High Altitude Medicine, People’s Liberation Army, Chongqing, China; ^4^Department of High Altitude Military Hygiene, College of High Altitude Military Medicine, Army Military Medical University, Chongqing, China

**Keywords:** HPC, neuroprotection, metabolomics, UHPLC-QTOFMS, metabolic pathway

## Abstract

**Background:** Hypoxia appears in a number of extreme environments, including high altitudes, the deep sea, and during aviation, and occurs in cancer, cardiovascular disease, respiratory failures and neurological disorders. Though it is well recognized that hypoxic preconditioning (HPC) exerts endogenous neuroprotective effect against severe hypoxia, the mediators and underlying molecular mechanism for the protective effect are still not fully understood. This study established a hippocampus metabolomics approach to explore the alterations associated with HPC.

**Methods:** In this study, an animal model of HPC was established by exposing the adult BALB/c mice to acute repetitive hypoxia four times. Ultra-high liquid chromatography-quadrupole time-of-flight mass spectrometry (UHPLC-QTOFMS) in combination with univariate and multivariate statistical analyses was employed to deciphering metabolic changes associated with HPC in hippocampus tissue. MetaboAnalyst 3.0 was used to construct HPC related metabolic pathways.

**Results:** The significant metabolic differences in hippocampus between the HPC groups and control were observed, indicating that HPC mouse model was successfully established and HPC could caused significant metabolic changes. Several key metabolic pathways were found to be acutely perturbed, including phenylalanine, tyrosine and tryptophan biosynthesis, taurine and hypotaurine metabolism, phenylalanine metabolism, glutathione metabolism, alanine, aspartate and glutamate metabolism, tyrosine metabolism, tryptophan metabolism, purine metabolism, citrate cycle, and glycerophospholipid metabolism.

**Conclusion:** The results of the present study provided novel insights into the mechanisms involved in the acclimatization of organisms to hypoxia, and demonstrated the neuroprotective mechanism of HPC.

## Introduction

Hypoxia appears in a number of extreme environments, including high altitudes, the deep sea, and during aviation, and occurs in cancer, cardiovascular disease, respiratory failures and neurological disorders. Brain tissue is very vulnerable to hypoxia that can cause headaches, dizziness, blurred vision, tinnitus, spatial learning and memory impairment, and even pathological changes (Davis and Hackett, 2017; Vetrovoy et al., 2017). Hypoxic preconditioning (HPC), motivated by the repetitive exposure of organisms, organs, tissues and cells to hypoxia is an intrinsic cytoprotection strategy, existing widely in the heart, brain, liver, kidney, and other organs (Dirnagl et al., 2009).

Hypoxic preconditioning is widely used in the study of anti-hypoxia nerve injury (Dirnagl et al., 2009; Rybnikova and Samoilov, 2015; Vetrovoy et al., 2017). Multiple studies have indicated that HPC could significantly protect the hippocampal tissue against hypoxic injury (Bickler et al., 2015; Gao et al., 2015; Saad et al., 2015). Additionally, the acute repetitive HPC mouse model has been widely employed to study animal behavior and intrinsic protection mechanisms under hypoxic conditions (Gao et al., 2006; Wang et al., 2008). A previous study demonstrated that the brain homogenates from preconditioned mice were able to strengthen the tolerance to hypoxia and protect the animals from hypoxic injury (Lu et al., 2005). Recent studies have examined the potential biomarkers of HPC (Hernandez-Jimenez et al., 2014; Lv et al., 2015). Cui et al. (2015) performed a proteomic study to profile the patterns of protein expression in HPC mouse brains. Although the protect effect of HPC has been confirmed, the underlying mechanisms remain unclear, especially at the endogenous metabolite level.

Metabolomics is a systemic biological approach, in which metabolic responses to environmental factors or physiological interventions are analyzed and modeled (Kell, 2004; Zamboni and Sauer, 2009; Madsen et al., 2010). Metabolomics provides an excellent prospect for discovering disease-specific metabolite signatures as putative biomarkers (Sreekumar et al., 2009). Metabolomics appears to be a promising approach to identifying metabolite-based biomarkers and revealing the underlying mechanism of neurodegenerative diseases (Jove et al., 2014), cardiovascular disease (Shah et al., 2012), and cancer (Richard et al., 2017). A previous study identified the molecular alterations associated with HPC mouse whole brains using metabolomics approach (Zhou et al., 2015). Recently, we have identified the molecular alterations associated with HPC mice serum using ultra-high liquid chromatography-quadrupole time-of-flight mass spectrometry (UHPLC-QTOFMS)-based metabolomics approach (Liu et al., 2017). This strategy exemplified the ability of metabolomics to identify endogenous biomarkers and elucidate the protective mechanism of HPC.

In this study, an acute repetitive HPC mouse model and a hippocampal metabolomic approach based on UHPLC-QTOFMS were established. One of the purposes was to identify the differential hippocampal metabolites in HPC associated with acute hypoxia and in normoxia controls. A further goal was to elucidate the mechanisms through which organisms acclimatize to hypoxia, in addition to the potential protective mechanism of HPC. This study deciphered HPC-related metabolites and metabolic pathways and provided novel insights into the neuroprotective mechanism of HPC.

## Materials and Methods

### Chemicals and Reagents

The assay kits for superoxide dismutase (SOD), malondialdehyde (MDA) and lactic acid were purchased from Jiancheng Bioengineering Institute (Nanjing, China). Formic acid was obtained from Fluka (Buchs, Switzerland). Acetone, citrate, and ammonium formate were obtained from Sigma-Aldrich (St. Louis, MO, United States). HPLC-grade methanol and acetonitrile (ACN) were purchased from Merk (Darmstadt, Germany). Valine, sodium succinate, phenylalanine, methionine, uric acid, arachidonic acid, linoleic acid, oleic acid, and palmitic acid were purchased from Shanghai Jingchun Reagent Co. Ltd. (Shanghai, China).

### Animals and Sample Collection

Male BALB/c mice, 6- to 8-week-old, weighing 18–22 g, were obtained from the Experimental Animal Center of Army Military Medical University. Mice were housed at 22 ± 2°C and 60 ± 10% relative humidity in a specific pathogen-free environment, with a 12/12 h light/dark cycle and *ad libitum* access to food and water. Thirty BALB/c mice were randomly divided into normoxic control (H0) group, acute hypoxic (H1) group, and acute repetitive hypoxia for four times (HPC) group.

Repeated hypoxic exposure were performed at room temperature (20 ± 1°C). The animal model of HPC was established according to our method described previously (Liu et al., 2017). After weighted, a mouse was placed in a 125-ml jar, which was sealed airtight with a rubber stopper and smeared with Vaseline. The mouse was taken out of the jar immediately following the appearance of the first asthmoid respiration (a sign of the hypoxia tolerance limit) (Wang et al., 2008); this was the first instance of hypoxia exposure. A maximum of 15 s was allowed for recovery under normoxic conditions. Subsequently, the mouse was immediately moved to another new jar with the same volume fresh in order to duplicate a progressive hypoxic environment three more times; the time of hypoxia tolerance in each mouse (from the beginning of the first airtight exposure to the final asthmoid respiration) was recorded. The H1 group was subjected to hypoxia only one time, and the H0 group did not undergo the hypoxic treatment. According to the following formula, the standard tolerance time was computed: *T* = *t*/(*v* -*w*)/0.94 × 100 (*T*, standard tolerance time; *t*, hypoxia tolerance time; *v*, jar volume; *w*, mouse weight).

At the end of the experiment, the mouse was sacrificed by decapitation. The hippocampus was isolated immediately on ice, frozen using liquid nitrogen, and stored at -80°C until analysis. All the animal experimental procedures and handling were approved by the Administrative Committee of Experimental Animal Care and Use of Army Military Medical University.

### Assessment of Antioxidative Activity and Anaerobic Metabolism in the Hippocampus

The antioxidative activity and the magnitude of anaerobic metabolism were detected with the levels of SOD, MDA, and lactic acid. All measurements were performed using the double antibody sandwich enzyme-linked immunosorbent assay (ELISA) kits according to the manufacture’s instructions.

### Sample Preparation

Each hippocampal tissues sample was weighed, and then homogenized by a bead mill homogenizer (Bullet Blender Blue, Next Advance). The tissue was transferred to a chilled safe-lock microcentrifuge tube. A mass of chilled stainless steel beads equal to the mass of the tissue was added to the tube. Ten-fold (w/v) ice-cold 80% methanol was then added to the tissue and beads. Samples were mixed in the Bullet Blender Blue for 2 min at a speed of eight. The extracts were centrifuged at 12,000 rpm for 10 min at 4°C, and all supernatant were lyophilized and then stored at -80°C until analysis. Samples were resuspended using 100 μl 80% methanol. In order to take into account the signal drift of the mass spectrometer over the run time, the hippocampus samples were injected into UHPLC-QTOFMS system in a random run order.

### UHPLC-QTOFMS Analysis

UHPLC-QTOFMS analysis was performed on Agilent 1290 Infinity LC system coupled with Agilent 6530 Accurate-Mass Quadrupole Time-of-Flight (Q-TOF) mass spectrometer. An acquity UHPLC HSS T3 C18 column (2.1 mm × 100 mm, 1.8 μm, Waters, Milford, Ireland) was used for hippocampal sample separation. The other detailed UHPLC-QTOFMS protocols were carried out according to our previously published paper (Liao et al., 2016).

### Data Handling

The data were pre-processed according to our previous work with slight modification (Liao et al., 2016). The raw data from UHPLC-QTOFMS were converted to mzData format by using Agilent MassHunter Qualitative software. XCMS package^1^ based R software was used to extract features in these data. The XCMS major parameters full width at half maximum (fwhm), bandwidth (bw), and snthresh were set as 10, 10, and 5, respectively. Other parameters in XCMS were default settings. The variables presenting in at least 80% of either group were extracted. The resulting dataset was normalized using the intensity of the internal standard, and then the ion peaks generated by the internal standard were removed. The resulting matrix (observation, retention time, m/z, intensity) were introduced into SIMCA-P 13.0 software (Umetrics, Umeå, Sweden) for principal component analysis (PCA) and partial least squares-discriminant analysis (PLS-DA), in which the data was mean centered and Pareto-scaled for both models. The quality of PCA and PLS-DA models was assessed with the relevant R2 and Q2 discussed elsewhere (Yin et al., 2009). One-way ANOVA was performed successively to reveal the statistical differences of the variables among different groups.

Metabolites identification was conducted according our previous work (Liao et al., 2016). Briefly, the quasi-molecular ion peak was identified according to ESI^+^ and ESI^-^ mode scans of UHPLC-QTOFMS. Then, the most probable molecular formula was calculated using the Agilent MassHunter software and the structure information were obtained by searching free databases such as Metlin, HMDB, and KEGG. Finally, the commercial standard MS/MS spectrum was used to confirm the identified compound. In addition, pathway analysis and visualization was performed by using web-based MetaboAnalyst 3.0 (Xia and Wishart, 2011; Xia et al., 2012).

## Results

### Animal Model of HPC

**FIGURE 1:**
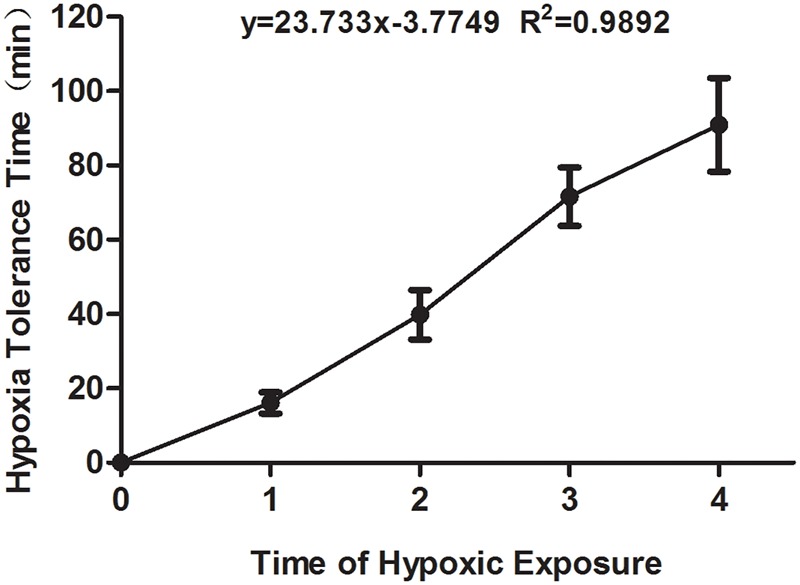
The hypoxia tolerance time of the experimental mice exposed to hypoxia from the first to fourth time. All comparison of each group showed the significant difference.

The time of hypoxia tolerance in mouse was recorded when the first asthmoid respiration appeared. As shown in Figure 1, the hypoxia tolerance of mice was prolonged with the increase in the time of hypoxia exposure, which suggested that the hypoxia preconditioned mouse model was successfully established.

### HPC Induced Enhanced Antioxidative Capacity in the Hippocampus Tissue

**FIGURE 2:**
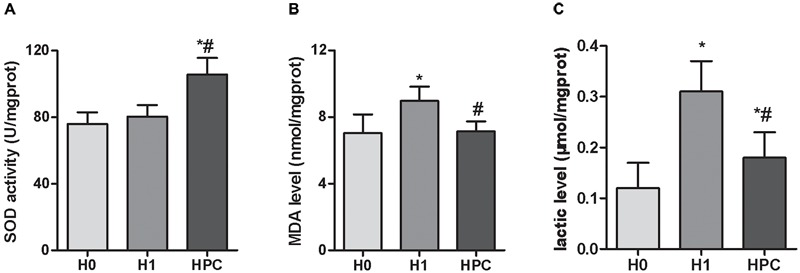
Effects of acute hypoxia and hypoxic preconditioning on the activity of SOD **(A)**, the contents of MDA **(B)**, and lactic acid **(C)** in the hippocampus of mice. Data are presented as mean ± SD. ^∗^*p* < 0.05 compared to H0 group. ^#^*p* < 0.05 compared to H1 group.

Compared with the H0 group, the hippocampal activity of SOD significantly increased in the mice in the H1 and HPC groups, while the levels of MDA and lactic acid significantly increased in the H1 group and decreased in the HPC group (Figure 2).

### Hippocampus Metabolic Profiling by UHPLC-QTOFMS

**FIGURE 3:**
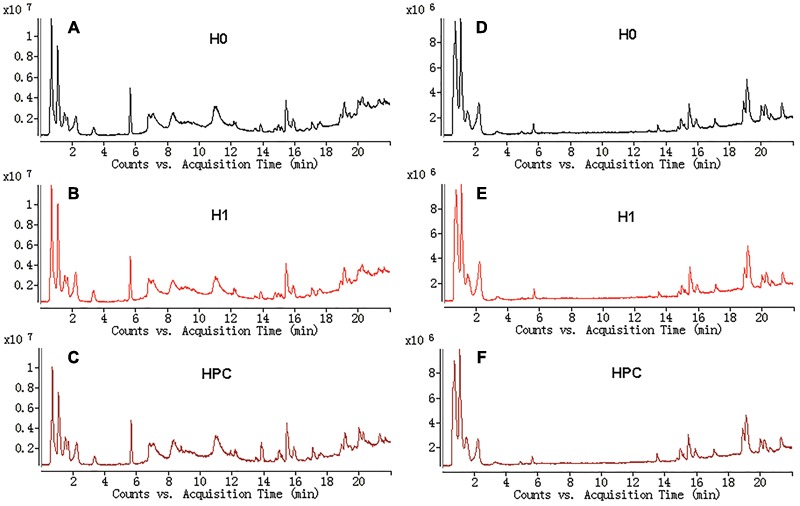
Typical total ions current chromatograms from hippocampus samples separated on LC ESI^+^ **(A–C)** and LC ESI^-^ **(D–F)**.

The typical total ion current (TIC) chromatograms of UHPLC-QTOFMS are shown in Figure 3. The stability of UHPLC-QTOFMS system is very important to obtain valid biochemical information. To assess the stability, the coefficients of variation (CV) of internal standard was calculated. It showed the CV was less than 15%, suggested that the method was robust. 1215 and 919 ion peaks were obtained from positive and negative ion modes of UHPLC-QTOFMS, respectively.

### Multivariate Statistical Analysis

**FIGURE 4:**
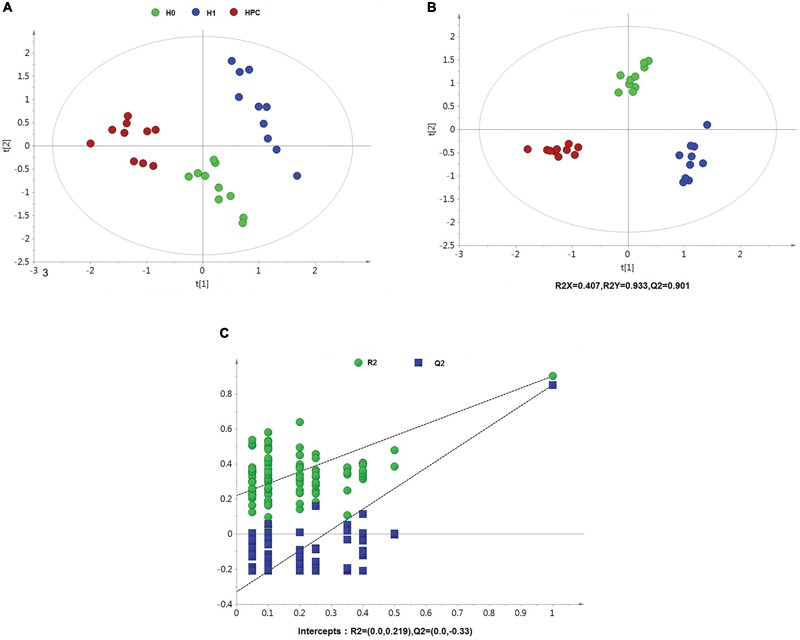
Multivariate data analysis based on hippocampus spectral data of UHPLC-QTOFMS positive ion mode. **(A)** PCA score map derived from UHPLC-QTOFMS spectra concerning H0, H1, and HPC groups. **(B)** PLS-DA score map derived from UHPLC-QTOFMS spectra concerning H0, H1, and HPC groups. **(C)** Validation plot obtained from 99 permutation tests.

**FIGURE 5:**
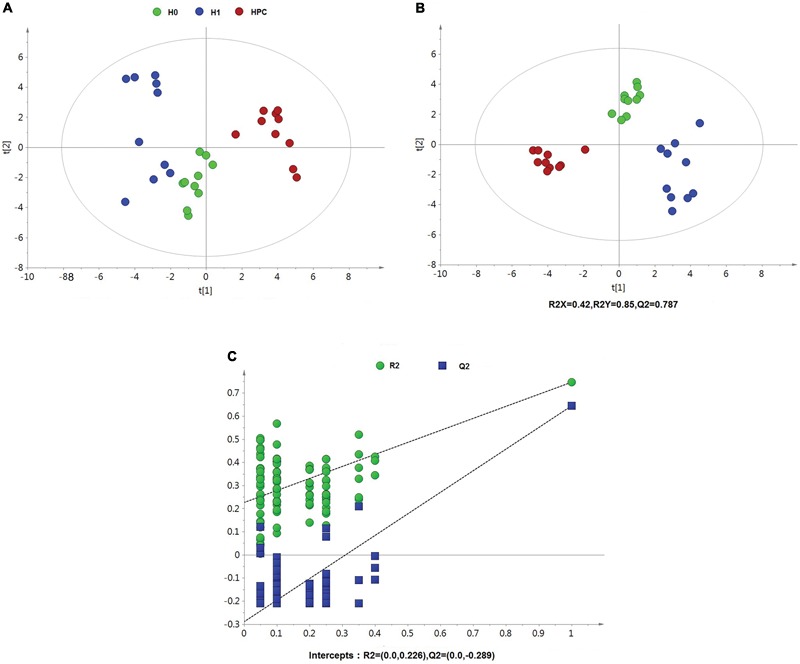
Multivariate data analysis based on hippocampus spectral data of UHPLC-QTOFMS negative ion mode. **(A)** PCA score map derived from UHPLC-QTOFMS spectra concerning H0, H1, and HPC groups. **(B)** PLS-DA score map derived from UHPLC-QTOFMS spectra concerning H0, H1, and HPC groups. **(C)** Validation plot obtained from 99 permutation tests.

After data normalization, an unsupervised PCA was conducted on the dataset, which a separated trend of inter-group was observed on the scores plots. The plots of unsupervised PCA and supervised PLS-DA scores obtained from the three groups were displayed in Figures 4, 5. The PLS-DA scores plots from positive and negative ion datasets showed that the H0, H1, and HPC groups could be separated distinctly. The PLS-DA models were validated by a permutation test (99 times). As shown in Figures 4C, 5C, model validation with permutation test generated intercepts of R2 = 0.219 and Q2 = -0.33 in positive ion dataset and R2 = 0.226 and Q2 = -0.289 in negative ion dataset, which meant that two the PLS-DA models were non-overfitting and reliable.

### Identification of Differential Hippocampus Metabolites in HPC

Metabolites of interest that significantly contributed to the separation of different groups were identified based on a threshold of variable importance in the projection (VIP) values (VIP > 1) generated from PLS-DA model. To select the potentially different metabolites, these discriminated metabolites were validated according to one-way ANOVA (*p* < 0.05). According to the above two different statistical methods, 34 significantly altered metabolites were identified in HPC (Table 1).

**Table 1:** Summary statistics and identifications of differentially expressed hippocampus metabolites and their metabolic pathways.

No.	tr/min	m/z	Adduct	Metabolite	VIP*^b^*	*P^c^*-value for ANOVA			Pathway involved
						H1 and H0	HPC and H0	HPC and H1	
**ESI(+)**									
1	0.70	258.109	M+H	Glycerophosphocholine	2.30	0.054	0.009	0.000	Glycerophospholipid metabolism
2	0.72	147.075	M+H	Glutamine*^a^*	2.76	0.000	0.003	0.001	Glutamine and glutamate
3	0.75	148.060	M+H	Glutamate*^a^*	1.64	0.000	1.000	0.000	Alanine, aspartate, and glutamate metabolism
4	0.80	146.117	M+H	Acetylcholine	1.62	0.000	0.670	0.000	Others pathway
5	1.59	182.081	M+H	Tyrosine*^a^*	1.36	0.000	1.000	0.000	Tyrosine metabolism
6	1.68	613.160	M+H	Oxidized glutathione	3.80	0.001	0.083	0.004	Glutathione metabolism
7	1.79	154.084	M+H	Dopamine*^a^*	1.26	0.000	0.000	0.000	Tyrosine metabolism
8	1.99	268.104	M+H	Adenosine*^a^*	1.82	0.010	0.000	0.000	Purine metabolism
9	3.37	166.086	M+H	Phenylalanine*^a^*	3.08	0.046	0.000	0.001	Phenylalanine metabolism
10	5.62	205.097	M+H	Tryptophan*^a^*	5.08	0.003	0.000	0.094	Phenylalanine, tyrosine, and tryptophan biosynthesis
11	14.14	300.290	M+H	Sphingosine*^a^*	1.63	0.001	0.000	0.000	Sphingolipid metabolism
12	15.17	424.343	M+H	Linoelaidylcarnitine	1.77	0.002	0.001	0.663	Fatty acid transportation
13	15.33	496.341	M+H	LysoPC (16:0)*^a^*	2.75	0.000	0.953	0.000	Glycerophospholipid metabolism
14	15.63	400.343	M+H	Palmitoylcarnitine	5.76	0.000	0.000	1.000	Fatty acid transportation
15	16.88	428.374	M+H	Stearoylcarnitine	1.56	0.000	0.000	1.000	Fatty acid transportation
16	17.11	524.373	M+H	LysoPC (18:0)*^a^*	2.65	0.000	0.874	0.001	Glycerophospholipid metabolism
17	20.27	808.584	M+H	PC (22:5/16:0)	1.75	0.000	0.000	0.051	Glycerophospholipid metabolism
**ESI(-)**									
1	0.68	124.007	M-H	Taurine*^a^*	1.74	0.000	0.000	0.000	Taurine and hypotaurine metabolism
2	0.70	102.056	M-H	γ-Aminobutyric acid	1.65	0.001	0.000	0.000	Alanine, aspartate, and glutamate metabolism
3	0.85	171.007	M-H	Glycerol 3-phosphate	2.99	0.013	0.001	1.000	Glycerolipid metabolism
4	0.94	168.991	M-H	Dihydroxyacetone phosphate	1.44	0.000	0.669	0.000	Glycolysis
5	1.10	179.056	M-H	Glucose*^a^*	1.76	0.000	0.050	0.000	Glycolysis
6	1.15	306.077	M-H	Glutathione	4.63	0.000	0.289	0.038	Glutathione metabolism
7	1.19	145.014	M-H	Oxoglutaric acid	1.41	1.000	0.071	0.009	Citric acid cycle
8	1.23	167.021	M-H	Uric acid*^a^*	1.22	0.000	0.005	0.008	Purine metabolism
9	1.28	147.030	M-H	2-Hydroxyglutarate	2.02	0.000	0.019	0.000	Others pathway
10	1.34	347.041	M-H	Inosine 5’-phosphate	9.35	1.000	0.000	0.000	Purine metabolism
11	1.41	191.020	M-H	Citric acid^a^	4.01	0.000	0.002	0.000	Citric acid cycle
12	1.47	151.026	M-H	Xanthine	3.01	0.017	0.000	0.118	Purine metabolism
13	1.54	117.020	M-H	Succinic acid*^a^*	4.60	0.456	0.000	0.003	Citric acid cycle
14	19.12	303.233	M-H	Arachidonic acid*^a^*	1.33	0.000	1.000	0.000	Arachidonic acid metabolism
15	19.30	279.233	M-H	Linoleic acid*^a^*	1.34	0.000	1.000	0.000	Linoleic acid metabolism
16	20.02	255.233	M-H	Palmitic acid*^a^*	1.54	0.000	0.772	0.000	Fatty acid metabolism
17	20.27	281.249	M-H	Oleic acid*^a^*	1.21	0.000	1.000	0.000	Fatty acid metabolism

*^*a*^Metabolites validated by reference standards. ^*b*^Variable importance in the projection (VIP) was obtained from PLS-DA with a threshold of 1.0. ^*c*^*P* means *p-*value obtained from ANOVA.*

### Metabolic Pathway Analysis

**FIGURE 6:**
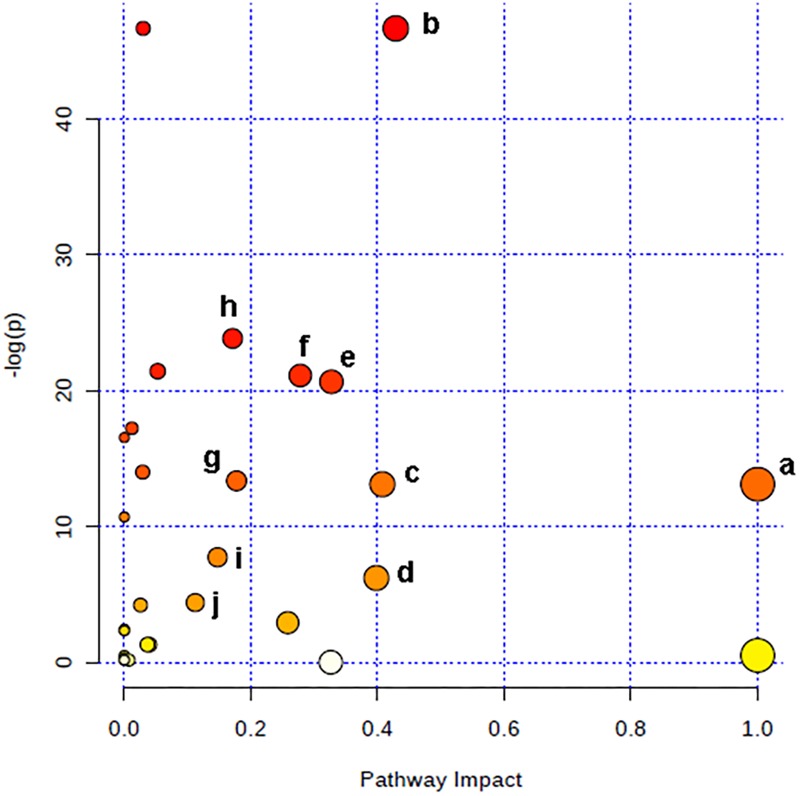
Altered metabolic pathways under HPC condition. a, phenylalanine, tyrosine, and tryptophan biosynthesis; b, taurine and hypotaurine metabolism; c, phenylalanine metabolism; d, glutathione metabolism; e, alanine, aspartate and glutamate metabolism; f, tyrosine metabolism; g, tryptophan metabolism; h, purine metabolism; i, citrate cycle; j, glycerophospholipid metabolism.

**FIGURE 7:**
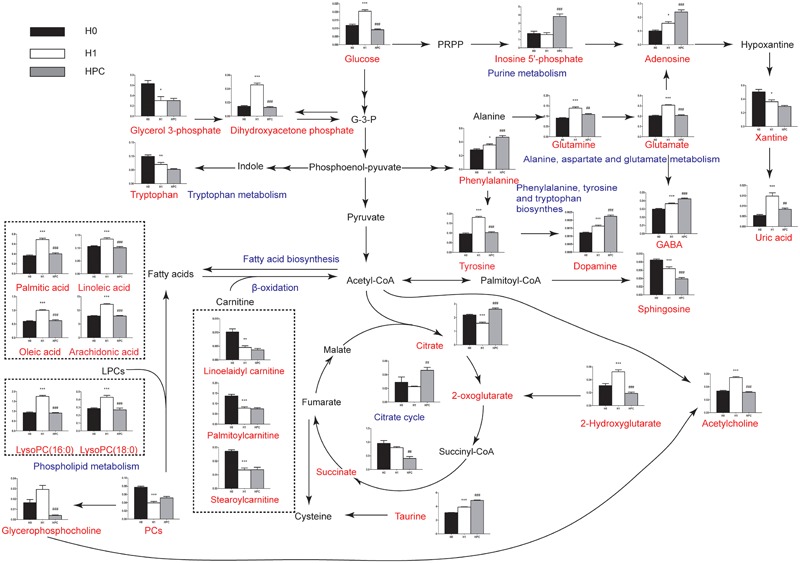
Schematic overview of the metabolites and major metabolic pathways. Red represents the detected metabolites, black represents undetected metabolites, blue represents the associated pathway, and the unrepresented indicated none detected in our experiment. ^∗^ represents the difference between H1 group and H0 group, ^∗^*p* < 0.05; ^∗∗^*p* < 0.01; ^∗∗∗^*p* < 0.001, # represents the difference between HPC group and H1 group, ^#^*p* < 0.05; ^##^*p* < 0.01; ^###^*p* < 0.001.

The relevant pathways and networks of HPC were revealed by MetaboAnalyst 3.0. According to a threshold of the impact-value (≥0.10), the potential target metabolic pathway of HPC were filtered, including phenylalanine, tyrosine and tryptophan biosynthesis, taurine and hypotaurine metabolism, phenylalanine metabolism, glutathione (GSH) metabolism, alanine, aspartate and glutamate metabolism, tyrosine metabolism, tryptophan metabolism, purine metabolism, citrate cycle, and glycerophospholipid metabolism were disrupted in HPC (Figure 6 and Table 2). In addition, further correlated pathways were constructed using the reference map obtained from KEGG (Figure 7).

**Table 2:** Result from metabolic pathway analysis with MetaboAnalyst 3.0^a^.

No.	Pathway name	Total cmpd	Hits	Raw *p*	-Log(*p*)	Impact
1	Phenylalanine, tyrosine, and tryptophan biosynthesis	4	2	2.04E-06	13.103	1.0000
2	Taurine and hypotaurine metabolism	8	1	5.53E-21	46.645	0.4286
3	Phenylalanine metabolism	11	2	2.04E-06	13.103	0.4074
4	Glutathione metabolism	26	2	2.02E-03	6.203	0.3979
5	Alanine, aspartate, and glutamate metabolism	24	4	1.09E-09	20.642	0.3270
6	Tyrosine metabolism	44	2	6.82E-10	21.106	0.2778
7	Tryptophan metabolism	40	1	1.59E-06	13.355	0.1772
8	Purine metabolism	68	5	4.48E-11	23.829	0.1710
9	Citrate cycle (TCA cycle)	20	3	4.40E-04	7.729	0.1472
10	Glycerophospholipid metabolism	30	3	1.24E-02	4.392	0.1120

*^*a*^Total is the total number of compounds in the pathway; the hits is the actually matched number from the user uploaded data; the raw *p* is the original *p-*value calculated from the enrichment analysis; the impact is the pathway impact value calculated from pathway topology analysis.*

## Discussion

Hypoxic preconditioning has been widely accepted as an endogenous protective effect. However, the underlying mechanism remains unclear. Here, using UHPLC-QTOFMS-based hippocampus metabolomics approach, 34 significant differential metabolites were identified. To understand the underlying biological functions of these metabolites, metabolic pathway analysis was conducted. The 34 metabolites were found to be involved in (a) phenylalanine, tyrosine and tryptophan biosynthesis, (b) taurine and hypotaurine metabolism, (c) phenylalanine metabolism, (d) GSH metabolism, (e) alanine, aspartate and glutamate metabolism, (f) tyrosine metabolism, (g) tryptophan metabolism and other three metabolic pathways. By relating these metabolic pathways, the regulated metabolic network of HPC-related potential biomarkers was established (Figure 7). The major metabolic patterns and plausible pathways are discussed in detail below.

### Increased Antioxidant Capacity

It is well-known that SOD is an important antioxidant and MDA is an index of free radial induced lipid peroxidation, which are commonly used as indicators of oxidative damage. The results of the present study demonstrated that, compared with the H0 group, the hippocampal activity of SOD significantly increased in the mice in the H1 and HPC groups, while the content of MDA significantly increased in the H1 group and decreased in the HPC group. These alterations indicated that the antioxidant capacity of hippocampal tissue was enhanced with the formation of HPC. Therefore, it was hypothesized that HPC protected the hippocampal tissue against hypoxic injury partially by enhancing the antioxidant capacity.

Glutathione, a main scavenger of free radicals in the body, can be divided into reductive GSH and oxidized GSH (GSSG). Under physiological conditions, GSH is essential to maintain cellular biological functions and to remove excess free radicals in the body (Enns and Cowan, 2017). The results of the present study demonstrated that GSH was significantly decreased in the H1 group and increased in the HPC group, whereas GSSG showed the opposite trend. The mechanism may be associated with the reduced activity of GSH reductase (promote the conversion of GSSG to GSH, using NADPH as substrate) induced by acute hypoxia (Maiti et al., 2008; Coimbra-Costa et al., 2017). This leads to a loss of antioxidant capacity of the GSH system and increased oxidative stress during acute hypoxia. After HPC, the level of GSH was increased and GSSG was decreased, indicating the increased antioxidant activity. We speculate that the regulation of GSH metabolism may serve a principal role in HPC. To increase GSH levels in the brain might be a potential strategy against hypoxia-induced brain injury.

Uric acid is generated by enzyme xanthine oxidase (XO) which catalyzes hypoxanthine to xanthine and then to uric acid (Sinha et al., 2009). The results of the present study indicated that the level of uric acid was significantly increased in the H1 group and decreased in the HPC group, while the level of xanthine was gradually decreased. The mechanism may be associated with the reduction in ATP levels when increased adenine nucleotide turn over was coupled with the stimulation of XO (Hare and Johnson, 2003). In addition, uric acid is an important non-enzymatic antioxidant. The decreased level of uric acid in the HPC group may indicate a decreased level of oxidative stress.

### Excitatory Amino Acid Content Was Significantly Reduced

Glutamate is a major excitatory neurotransmitter working at a variety of excitatory synapses in the nervous system and excessive extracellular glutamate concentrations are neurotoxic (Rothman and Olney, 1995). The results of the present study indicated that the level of glutamate was significantly increased in the H1 group and decreased in the HPC group, whereas the inhibitory neurotransmitter, including γ-aminobutyric acid (GABA), dopamine and adenosine, and taurine as the antagonist of excitatory amino acid gradually increased with the formation of HPC. Therefore, it was hypothesized that HPC ameliorates excitotoxicity partially by shifting from excitatory glutamate-mediated neurotransmission to inhibitory GABA-mediated neurotransmission (Dave et al., 2005; Yenari et al., 2008) and/or upregulating the levels of dopamine (Kim et al., 2004) and adenosine. Additionally, previous studies have demonstrated that taurine modulated neuronal activity, combated ER stress responses induced by glutamate or regulated the balance of intracellular and extracellular calcium ions, and protected neurons from ischemic and hypoxic injury, in addition to other functions (Tang et al., 2014; Prentice et al., 2017). Therefore, upregulating the abovementioned inhibitory neurotransmitter might be beneficial to the tolerance of mice to hypoxia.

### Altered Energy Metabolism

The level of lactic acid was increased in HPC-induced mice, which suggested that anaerobic glycolysis was involved in HPC. Meanwhile, the levels of citrate and oxoglutaric acid were increased, which suggested that mitochondrial TCA cycle activity was inhibited in the HPC group. Reportedly, the activity of lactate dehydrogenase (LDH) was significantly increased and succinate dehydrogenase (SDH) was decreased in the hippocampus of the hypoxia-preconditioned mice. This result indirectly substantiated the results of the present study (Lu et al., 2005).

Inosine 5′-phosphate is a key substrate of the *de novo* synthesis of guanine nucleotides, which is catalyzed to xanthosine 5′-phosphate by inosine-5′-phosphate dehydrogenase with the nicotinamide adenine dinucleotide (NAD^+^)-dependent oxidation (inosine 5′-phosphate + NAD^+^ + H_2_O ⇌xanthosine 5′-phosphate + NADH + H^+^). This process plays an important role in regulating the synthesis of DNA and RNA (Greenberg, 1951). Our data showed that the inosine 5′-phosphate was significantly increased and NADH was decreased in the HPC group, which indicated that the DNA and RNA synthesis were reduced with the formation of HPC. The purpose of these changes may be to reduce the energy consumption.

### Altered Lipid Metabolism

Our results indicated that lysophosphatidylcholines (LysoPCs) [(LysoPC (16:0) and LysoPC (18:0)] and free fatty acids (FFAs) (arachidonic acid, linoleic acid, oleic acid, and palmitic acid) are significantly increased in the H1 group and decreased in the HPC group, whereas PC demonstrated the opposite trend. These alterations may be putatively ascribed to the activation of phospholipase A2 (PLA2), which mediates the release of lysoPCs and specific fatty acids from PC. Reportedly, PLA2 was significantly increased under hypoxia, whereas decreased gradually to a normal level with the formation of HPC. This result indirectly substantiated the results of the present study (Liu et al., 2006). Our study indicated that the phospholipid metabolic pathway may serve a principal role in HPC.

Our result demonstrated that sphingosine was significantly decreased in the H1 and HPC groups, with the lowest levels in the HPC group. It is conceivable that this alteration may be ascribed to the activation of sphingosine kinase (SphK), which catalyzes the conversion of sphingosine to sphingosine phosphate-1. Reportedly, the activity of SphK2 was significantly increased with the formation of HPC. This result indirectly substantiated the result of the present study (Wacker et al., 2009, 2012). Thus, we speculate that the sphingolipid metabolic pathway may play an important role in HPC and the bioactive components of this pathway may be suitable preventive and therapeutic targets for protecting the brain tissue against hypoxic injury.

## Conclusion

In conclusion, an UHPLC-QTOFMS-based hippocampus metabolomic approach was developed to profile the HPC-related metabolic alterations. Our results demonstrated that the HPC mouse model was well-established. In the current study, 34 HPC-related metabolites in hippocampus and the HPC-regulated metabolic network were identified. The identified target metabolites were found to encompass a variety of process associated with oxidative stress, excitatory amino acid and energy metabolism. The results provided a new insight into the neuroprotective mechanism of HPC and suggested that modifying these points of convergence is a promising approach for the treatment of hypoxia-related diseases.

## Author Contributions

W-TL, W-YL, and Y-QG conceived and designed the experiments. JL, S-MZ, and GX performed the experiments. W-TL and JL analyzed the data. W-TL wrote the paper. All authors contributed to the interpretation of results, critical revision of the manuscript, and approved the final manuscript. W-YL is the guarantor.

## Conflict of Interest Statement

The authors declare that the research was conducted in the absence of any commercial or financial relationships that could be construed as a potential conflict of interest.

## Footnotes

1 https://metlin.scripps.edu/landing_page.php?pgcontent=mainPage
